# Targeting estrogen receptor signaling for treating heart failure

**DOI:** 10.1007/s10741-023-10356-9

**Published:** 2023-10-03

**Authors:** Chenyue Qian, Jingjin Liu, Huadong Liu

**Affiliations:** 1 The Second Clinical Medical College, Jinan University, Shenzhen, 518020 Guangdong China; 2 Department of Cardiology, Shenzhen People’s Hospital (The Second Clinical Medical College, Jinan University, The First Affiliated Hospital, Southern University of Science and Technology), Shenzhen, 518020 Guangdong China; 3 Department of Cardiology, Shenzhen Cardiovascular Minimally Invasive Medical Engineering Technology Research and Development Center, Shenzhen People’s Hospital (The Second Clinical Medical College, Jinan University, The First Affiliated Hospital, Southern University of Science and Technology), Shenzhen, China; 4 Shenzhen Key Laboratory of Stem Cell Research and Clinical Transformation, Shenzhen People’s Hospital (The Second Clinical Medical College, Jinan University, The First Affiliated Hospital, Southern University of Science and Technology), Shenzhen, 518020 Guangdong China; 5 Department of GeriatricsThe Second Clinical Medical CollegeThe First Affiliated Hospital, Shenzhen People’s HospitalJinan UniversitySouthern University of Science and Technology), Shenzhen, 518020 Guangdong People’s Republic of China

**Keywords:** Heart failure, Estrogen, Estrogen receptor, Hormone replacement therapy, Cardiac rehabilitation

## Abstract

Heart failure (HF) is a significant public health problem worldwide. It has long been noted that premenopausal women, compared to postmenopausal women and men, have lower rates for developing this disease, as well as subsequent morbidity and mortality. This difference has been attributed to estrogen playing a cardioprotective role in these women, though exactly how it does so remains unclear. In this review, we examine the presence of estrogen receptors within the cardiovascular system, as well as the role they play behind the cardioprotective effect attributed to estrogen. Furthermore, we highlight the underlying mechanisms behind their alleviation of HF, as well as possible treatment approaches, such as hormone replacement therapy and exercise regimens, to manipulate these mechanisms in treating and preventing HF.

## Introduction

Cardiovascular disease continues to be a major cause of mortality worldwide [1]. One of its most significant manifestations is heart failure (HF), stemming from structural and/or functional cardiac defects [2]. However, it has long been noted that premenopausal women have lower cardiovascular disease risk, compared to aged-matched males, which may be owed, at least in part, to the presence of sex hormones like estrogen [3]. This is further supported by observations of HF being more highly prevalent among postmenopausal women [4]. As a result, sex hormones have been considered to possibly play key roles in protecting against HF development [5], and substantial research has been conducted on the role of estrogen in cardiovascular disease etiology [6].

Estrogens have been found to exert a variety of beneficial effects on the cardiovascular system, such as antioxidative [5] and anti-inflammatory [7] activities, as well as being able to prevent atherogenesis [8], thrombosis [8], and cell proliferation [2]; it exerts cardiovascular protective effect mainly by inhibiting the proliferation of vascular smooth muscle cells [9]. All of these findings thus point to estrogen potentially serving as a cardioprotective agent, via its ability to alter cardiovascular physiology and function in health and disease. In this review, we examine the literature regarding the molecular bases of estrogen and its receptor in counteracting against HF and how their dysregulation results in disease development. This research, in turn, provides a foundation for developing therapies to treat HF.

## The role of 17-β-estradiol (E2) and its receptors in the cardiovascular system

The most common form of circulating estrogen is 17-β-estradiol (E2), whose effects on the cardiovascular system are mediated through nuclear and membrane estrogen receptors (ERs), such as ERα, ERβ, and G-protein-coupled ER (GPR30/GPER) [10]. One process mediated by E2 binding on ERs is nitric oxide (NO) production in cerebral and peripheral endothelial cells, through endothelial NO synthase (eNOS) [10]. NO is a key vasodilator and cardio-protective factor, being able to maintain blood vessel dilation, regulate blood pressure, and activate a variety of vaso-protective and anti-atherosclerotic processes in vascular endothelial cells [11]. By contrast, NO and cyclic guanosine phosphate (cGMP) pathways are downregulated in postmenopausal women, owing to the activation of the renin–angiotensin–aldosterone system (RAAS) in response to lowered estrogen levels. These women have been characterized as possessing more rigid arteries and cardiac muscles, rendering them less sensitive to aberrant blood flow loads [12].

### ERα

Among HF individuals with preserved ejection fraction (HFpEF), right ventricular (RV) dysfunction is a major predictor of mortality [13]. E2 has been documented to play a protective role in maintaining RV function and counteracting against maladaptive remodeling, via ERα. Investigations of the underlying mechanisms revealed that E2-ERα binding activated bone morphogenetic protein receptor 2 (BMPR2) signaling, which has been identified as a potent effector of cardiac contractile force [14, 15]. Previously, BMPR2, along with the peptide hormone Apelin, have been noted to be required for cardiac development and pulmonary vascular homeostasis [16]. Newer evidence, though, has indicated that the E2-ERα-BMPR2 pathway plays a role in maintaining RV function, serving as the molecular basis for the cardioprotective effect of E2 there [17]. This is further supported by ERα slowing down RV remodeling among female rats with pulmonary hypertension [7], as well as by Cheng et al., who induced RV hypertrophy, via pulmonary artery banding (PAB), among male rats, as well as both wild-type (WT) and ERα loss-of-function mutant females [18]. They discovered that mutant females, compared to WT and males, had diastolic dysfunction and higher collagen type I to III ratios, indicating that ERα signaling in females defended against collagen buildup and diastolic dysfunction during the RV pressure overload response. Therefore, the protective effect of E2-ERα against diastolic dysfunction is sex-specific [18].

### ERβ

ERβ has also been identified as playing cardioprotective roles in recent years [19–21]. For instance, ovariectomized (OVX) and ERβ-deleted mice showed aberrant vascular function, hypertension, higher death rates, and worsened HF [22]. Furthermore, ERβ has been observed to exert anti-hypertrophic and anti-fibrotic actions among both OVX and non-OVX mice [21, 23, 24]. ERβ-deleted mice, compared to WT, also have been found to exhibit significant increases in inflammatory pathway activation, when the heart is subject to transverse aortic constriction (TAC)-induced hypertrophy [25]. By contrast, the presence of ERβ was able to attenuate cardiac remodeling and apoptosis in the TAC mouse model, according to Fliegner et al. [26]. Additionally, ERβ was determined to be essential for controlling the proteome response to pressure overload, which might be a key factor in delaying the beginning of HF [27]. Following these findings, several unbiased studies also showed that ERβ activation was responsible for some of those anti-apoptotic effects attributed to E2 and sex difference. In accordance with these findings, Cao et al. [28] showed that E2 treatment in vivo reduced cardiac rupture likelihood among myocardial infarcted (MI) male mice, which was accompanied by decreased matrix metalloproteinase-9 (MMP-9) activation and increased anti-apoptotic Bcl-2, compared to un-infarcted controls. E2 treatment was also found by Pedram et al. to prevent angiotensin II (Ang II)-induced cardiac hypertrophy, a precursor to diastolic stiffness, among female mice. Additionally, Ang II-induced myosin heavy chain synthesis, ERK activation, calcineurin activity, and interstitial fibrosis were all inhibited. Such prevention was only present among WT or ERα-null mice, not for ERβ-null, reinforcing that E2 operated through ERβ to exert its cardioprotective effects [29]. All these findings thus suggest that ERβ could serve as a potential therapeutic target against HF, which is further supported by studies investigating ERα and ERβ agonists, in which ERβ was found to be the main receptor for E2 being able to rescue cardiac functioning in HF [30].

Aside from its cardioprotective role, ERβ activation is also a prerequisite for estrogen-dependent upregulation of both eNOS and inducible NOS (iNOS) in rat neonatal cardiomyocytes [31]. eNOS, as well as neuronal NOS (nNOS) activities, have significant impacts on diastolic function, in which nNOS inhibition enhanced diastolic function in OVX rats, according to Jessup et al. [32] Tetrahydrobiopterin, a NOS cofactor, is hypothesized to be activated by E2 in order to control NOS production. Therefore, E2 deficiency in OVX rats leads to less activated tetrahydrobiopterin being available, which may have caused nNOS uncoupling, resulting in them shifting from catalyzing NO formation to generating superoxides [32]. This increase in nNOS-produced superoxides and decrease in proper NOS metabolites may result in diastolic stiffness, impaired cardiac remodeling, and eventually HF.

#### GPR30

G-protein-couped receptors (GPCRs) are the main membrane receptor class involved in mediating the effects of cardiac disorders [33]. One of those GPCRs is GPR30/GPER, which has been found to exert positive impacts on female HF patients [34], via its engagement in non-genomic estrogen signal transduction in nervous, reproductive, skeletal, immune, and cardiovascular systems, as well as in metabolism [35]. For instance, G1, a GPR30 agonist, was able to lower Ang II-induced hypertrophy among neonatal cardiomyocytes, via stimulating the upregulation of the PI3K-Akt-mTOR signaling pathway and inhibiting autophagy [36, 37]. GPR30, like ERβ, also appears to play a cardioprotective role against oxidative stress, as evidenced by the finding that a mitochondria-targeted antioxidant was able to reduce cardiac oxidative stress, which was otherwise elevated, among female animals possessing cardiomyocyte-specific mutant GPR30 [38]. This is further supported by the fact that G1 lowered cardiac atrial (ANP) and brain natriuretic peptides (BNP), as well as myosin heavy chain (MHC) levels, in Ang II-induced cardiac hypertrophy rats. With respect to Ang II stimulation, reactive oxygen species (ROS) are produced when it binds to the Ang II type 1 receptor (AT1R) [39, 40], which eventually leads to HF by increasing oxidative stress, hypertrophy, and apoptosis [41]. On the other hand, G1 administration suppressed cardiac fibrosis, apoptosis, and oxidative stress, demonstrating that GPR30 activation was able to counteract against cardiac remodeling [42], which was further proven by Da Silva et al. [43], who discovered that G1 administration was necessary for improving diastolic function among spontaneously hypertensive OVX rats. Chronic G1 treatment was also found to enhance aortic ring reactivity to acetylcholine by lowering cardiac angiotensin-converting enzyme activity, AT1R protein expression, and Ang II immunoreactivity[44].

To further examine the role of GPR30 in cardiomyocytes, a cardiomyocyte-specific KO animal model was developed by Wang et al. [43]. There, they found that GPR30 KO had diastolic dysfunction, as well as other cardiovascular disease-associated traits, thereby proving that GPR30 may be necessary for maintaining overall cardiac function. An isoproterenol-induced HF model was also examined, in which the progression of left ventricular (LV) cardiomyocyte dysfunction, with significantly decreased dL/dtmax, dR/dtmax, and [Ca^2+^]i, as well as β-adrenergic receptor (AR) desensitization and maladaptive remodeling in terms of their shapes, was paralleled by LV chamber abnormalities. All of these changes, however, were reversed towards normal levels after G1 treatment, which could be due to the normalization of basal and β-AR-stimulated Ca^2+^ handling, leading to the reversal of cardiomyocyte relaxation and force generation abnormalities stemming from HF. Therefore, G1 could serve as a potential therapeutic approach to counteract against HF, via restoring normal [Ca^2+^] regulation [34]. This is further supported by reports of HF, or GPR30 deficiencies, both leading to reduced LV SERCA2a expression and activity [45–47], along with increased sarcolemmal Na^+^-Ca^2+^ exchange, sarcoplasmic reticulum Ca^2+^ leakage, and faulty Ca^2+^ removal. On the other hand, GPR30 increases SERCA2a expression and activity to bolster myocardial Ca^2+^ mobilization [47, 48], via reversing HF-induced alterations in cardiac β1- and β2-AR expression and activity. Overall, data suggests that elevated oxidative stress leads to cellular damage, defective [Ca^2+^]i control, and remodeling during HF, whereas GPR30 activation was able to prevent cardiomyocyte apoptosis and unfavorable LV remodeling [45, 47]. Therefore, GPR30 could possibly counteract against HFpEF, by restoring cardiac β-AR responsiveness, as well as counteracting against LV and cardiomyocyte contractile abnormalities [34] (see Table 1).

**Table 1:** Overview of the different types of estrogen receptors (ER) and their roles in the cardiovascular system

ER	Mechanisms involved and outcomes	References
ERα	Activates bone morphogenic protein receptor 2 signaling	[14, 15, 17, 18]
ERβ	-Responsible for the anti-hypertrophic, anti-fibrotic, and anti-apoptotic activities associated with 17-β-estradiol -Controls cardiac proteome response to pressure overload	[21, 23–30]
GPR30	-Selective agonist G1 is able to reduce angiotensin II-induced hypertrophy among neonatal cardiomyocytes -Counteracts against oxidative stress, apoptosis, and adverse left ventricular remodeling -Lowers cardiac atrial natriuretic peptide, brain natriuretic peptide, and myosin heavy chain levels -Reduced myocardial hypertrophy and fibrosis	[34–42, 44–48]

## Pharmacological therapy

The beneficial effects of estrogen/ER activation on cardiac functioning in HF have resulted in the development of numerous treatment approaches. One possible approach is to upregulate myocardial cGMP signaling, as examined in the Vericiguat Global trial in Subjects with Heart Failure and Reduced Ejection Fraction (VICTORIA), where it was found that the soluble guanylate cyclase (sGC) stimulant, vericiguat, exerted cardioprotective effects [49]. As lowering estrogen levels have been associated with lowered cGMP, it is thus reasonable to speculate that similar approaches to the VICTORIA trial could be used for activating estrogen/ER signaling in HF. Indeed, the myocardial cGMP-PKG signaling pathway has been found to be deactivated in HFpEF, which has been found to occur among females independently of obesity and diabetes. Furthermore, among female mice, cGMP-PKG activation among cardiomyocytes by the phosphodiesterase type 5 (PDE5) inhibitor sildenafil was able to alleviate HF [50, 51], which, however, requires estrogen signaling to activate eNOS-dependent cGMP and PKGIα [51]. More specifically, it involves a nonnuclear signaling mechanism, triggered by ER binding to striatin; this is blocked, though, in ER mutants [52].

However, discrepancies in the outcomes of clinical studies comparing hormone replacement therapies (HRT) for treating HF have been noted [53]. This may be due to cellular and organ functioning among postmenopausal women with cardiovascular disease being affected by the complex hormonal environment. Exogenous substances, such as the progesterone endocrine disruptor medroxyprogesterone acetate, as well as conjugated estrogen from horse urine, failed to exhibit the same positive effects as endogenous hormones [54]. Furthermore, they were associated with significant negative side effects, such as increased blood clotting and inflammation, compared to transdermal estradiol [55]. Therefore, the timing for the initiation of hormone therapy, as well as the agents used, are likely factors behind its failure to achieve beneficial cardiac effects [56].

Despite the failures in hormonal therapy, though, estrogen has been found to improve cardiovascular-associated indices, such as exercise endurance and arterial NO-dependent dilation, as well as slowing down subclinical atherosclerosis development. It is also crucial, in terms of diastolic function, for lowering isovolumetric relaxation time and raising the E/A ratio. Therefore, we believe that the timing and route of estrogen administration may be essential for obtaining its beneficial effects. This was supported by evidence showing transdermal administration of estrogen balanced out its benefits and side effects, transdermal delivery prevents the cardiovascular thromboembolic damage associated with oral oestrogen, while oral administration was associated with some negative cardiovascular effects [57]. The use of oral estrogens (diethylstilbestrol) increases thromboembolic cardiovascular disease [58]. The “timing hypothesis” postulates that the recipient’s age and hormonal environment affect the effect of E2 injection on the vasculature [59, 60]. Age has been proven to be a significant factor in determining the vascular effects of E2 in women, with positive effects being seen in younger (60 years) postmenopausal women but not in older (> 60 years) postmenopausal women [61]. Hodis et al. [62] discovered that the effects of estradiol (with or without progesterone) on the development of atherosclerosis varied depending on the timing of therapy initiation, with benefit noted when it was started in women who were less than 6 years past menopause but not in those who were 10 or more years past menopause. Therefore, the finding that estrogen administration during early menopause has more beneficial cardiovascular effects [62, 63], compared to other time points, suggests that future estrogen therapy approaches may be most successful if applied at younger ages. In fact, Gersh et al. suggested that if HRT was required, it should be started right away after the cessation of ovarian hormone production, and administered as transdermal estradiol, in conjunction with cyclic dosing of human-identical progesterone [64].Therefore, utilizing the proper timing, administration method, and formulation for estrogen replacement therapy may maximize its benefits and minimize its side effects. At present, the existing clinical data on the timing and pathway of estrogen administration are insufficient, which may provide a possible new approach for clinical research on estrogen receptor activation, which still needs to be further studied.

## Exercise therapy

It is worth noting that reaching the recommended 150 min/week of physical activity is more difficult for female patients than for men because they face multiple barriers, such as time constraints caused by family or work responsibilities and a feeling that corporate responsibility is either tedious or inappropriate for young people. Therefore, to address increased cardiovascular disease risk among postmenopausal women, which may not be addressed by standard CR, alternative, cutting-edge exercise therapies must be investigated [65]. In fact, female congestive HF patients are able to dramatically increase their fitness level with CR, and actually exhibit greater improvements in tests like the 6-min walk test (6MWT), compared to male patients. Additionally, similar findings imply that exercise capacity, in the form of 6-min walk distance (6MWD), self-reported exercise levels, and mood (CDS scores) are able to simultaneously improve over time in female HF patients [66]. Aerobic, as well as resistance exercise, has also been found to lower pro-inflammatory biomarkers among obese postmenopausal women, who had lower levels of IL-2, IL-4, IL-6, and TNF-α [67]. This may be due to it counteracting the effects of increased adiposity and altered lipid profiles, stemming from hormonal changes, among postmenopausal women [68] (see Table 2).

**Table 2:** Therapies to activate the estrogen-receptor axis to treat heart failure

Therapy types	Mechanism	Treatment regimen	References
Pharmacological	cGMP signaling upregulation	Sildenafil (phosphodiesterase type 5 inhibitor)	[50–52]
	Hormone replacement therapy	-Transdermal estradiol, in conjunction with cyclic dosing of human-identical progesterone -Initiated immediately after the cessation of ovarian hormone production	[53–56]
Exercise	-Reduced sympathetic autonomic drive -Increased vagal autonomic drive -Lowered levels of interleukin (IL)-2, -4, -6, and tumor necrosis factor-α	-Moderate-to-vigorous intensity continuous exercise -High-intensity interval training	[65–68]

## Conclusion

In conclusion, the stimulation of estrogen receptors, particularly through the administration of a specifically arranged HRT regimen, or via cardiac rehabilitation exercises, could serve as a possible treatment approach for improving cardiac functioning post-HF, especially among postmenopausal females. However, these treatment regimens should be tailored for the needs of each patient.

## Data Availability

The data that support the findings of this study are available from the corresponding author, Dr. Jingjin Liu, upon reasonable request.

## Abbreviations

HF: Heart failure
HFrEF: Heart failure with decreased ejection fraction
GPR30/GPER: G-protein-coupled ER
